# Successful emergency endovascular aortic repair for intratumoral hemorrhage in extensive retroperitoneal mass of testicular origin

**DOI:** 10.1186/s12893-020-00933-2

**Published:** 2020-11-07

**Authors:** S. Hulova, R. Aziri, I. Vulev, P. Palacka, G. Kolnikova, K. Rejlekova, M. Chovanec, J. Mardiak, D. Pindak, M. Mego

**Affiliations:** 1 Department of Oncology, Central Military Hospital SNP Ruzomberok, Ruzomberok, Slovak Republic; 2 Department of Surgical Oncology, Slovak Medical University, Bratislava, Slovak Republic; 3 Center of Interventional Neuroradiology and Endovascular Treatment, Bratislava, Slovakia; 4 2nd Department of Oncology, Faculty of Medicine, National Cancer Institute, Comenius University, Klenova 1, 833 10 Bratislava, Slovak Republic; 5 Department of Pathology, Slovak Medical University, Bratislava, Slovak Republic; 6 National Cancer Institute, Bratislava, Slovak Republic

**Keywords:** Endovascular repair, Advanced testicular cancer, Aortic rupture, Retroperitoneal tumor, Case report

## Abstract

**Background:**

Metastatic germ cell cancer of the testis is characterized by favorable prognosis since effective treatment methods are available even in cases of extensive disease. Retroperitoneal masses frequently encroach major blood vessels requiring a vascular intervention usually performed in association with the post-chemotherapy retroperitoneal lymph node dissection (RPLND). Reported clinical case describes a successful pre-treatment endovascular surgery for abdominal aortic rupture allowing for full-dose systemic chemotherapy administration, and subsequent radical surgical intervention at primary tumor site as well as metastatic retroperitoneal lymph node dissection including the reconstruction of inferior caval vein.

**Case presentation:**

Patient presented with left-sided testicular tumor and voluminous retroperitoneal mass with vascular involvement. Soon after the patient had been admitted for the first cycle of cisplatin-based chemotherapy, computed tomographic angiography (CTA) revealed a dorsal aortic wall rupture with active extravasation and irregular pseudoaneurysmatic dilatation of the aorta below the leak area. Retroperitoneal intratumoral hemorrhage associated with the bilateral iliac venous thrombosis required an endovascular repair procedure of infrarenal abdominal aorta.

**Conclusions:**

Following the successful endovascular aortic repair 3 cycles of BEP (bleomycin, etoposide, cisplatin) regimen were administered with subsequent delayed left radical orchiectomy and RPLND associated with vena cava inferior (VCI) resection. Reconstruction of VCI was originally not deemed necessary as collateral blood flow appeared sufficient, however, intraoperative complications resulted in the need for unilateral VCI reconstruction, using the interposed bypass between right common iliac vein and infrarenal segment of VCI. Histopathologic examination of the attained specimen detected no vital cancer structures. The patient remains disease-free 18 months after the RPLND.

## Background

Testicular germ cell tumors often present with advanced stages including extensive retroperitoneal disease which might encroach the vital vasculature. Cisplatin-based chemotherapy combined with radical surgery provides excellent disease control [1] while incomplete resection of residual masses significantly reduces the long-term survival [2].

Vascular reconstructive surgery is often a part of planned surgical intervention in the management of residual masses in the post-chemotherapy period [3, 4]. The probability of vascular procedure in patients with intermediate or poor risk classified by The International Germ Cell Cancer Collaborative Group (IGCCCG) classification system and residual disease > 5 cm is around 20% [5]. Only few cases of bleeding events secondary to metastatic testicular cancer were reported to date, including aortic pseudoaneurysm, aortoenteric fistulas in the duodenum or stomach and intraabdominal hemorrhage from hepatic necrosis after the chemotherapy [6–9].

Herein we present a patient with newly diagnosed lefd-sided testicular tumor, bulky retroperitoneal tumor involving the major blood vessels along with the aortic wall rupture and active leakage what urged a vascular intervention prior to systemic therapy.

## Case presentations

**Fig. 1:**
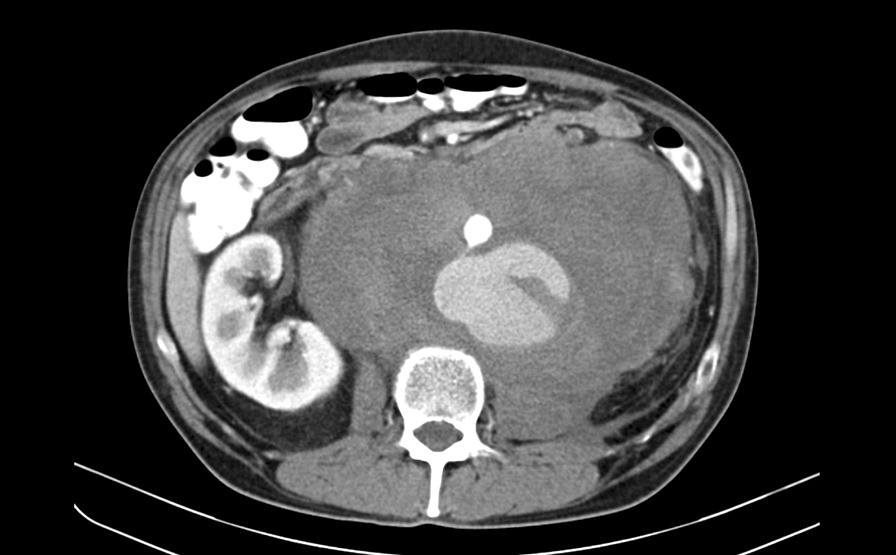
CT scan showed active extravasation of contrast agent into retroperitoneal tumor mass. ( transversal view)

**Fig. 2:**
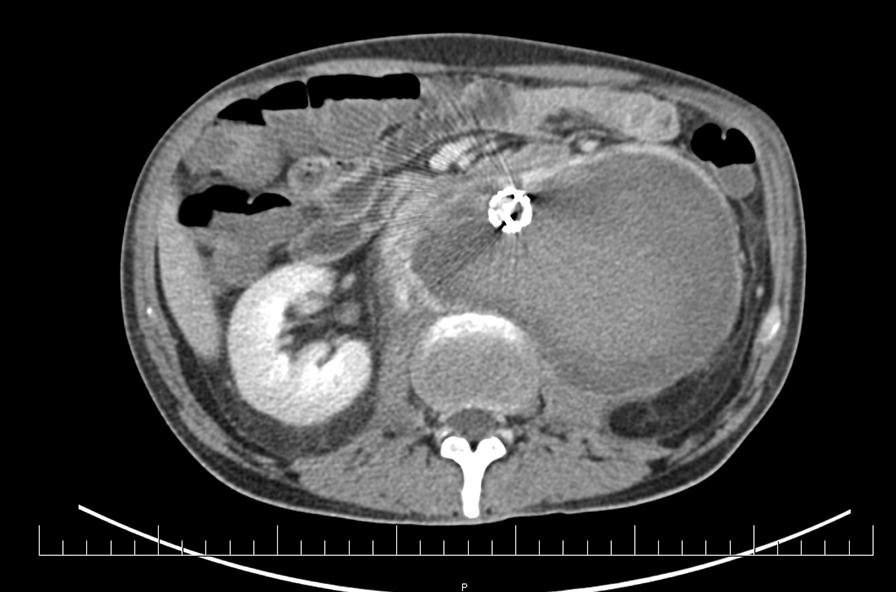
Two Endurant abdominal stentgrafts were successfully implanted via right common femoral artery

A 48-year-old patient with personal history of arterial hypertension and toxic hepatopathy was admitted to the National Cancer Institute in Bratislava, presenting with a newly diagnosed left-sided testicular tumor and a 215 × 145 × 170 mm (Cranio-Caudal × Latero-Lateral × Antero-Posterior) (CCxLLxAP) heterogeneous retroperitoneal mass, compressing the inferior caval vein and left ureter, with bilateral iliac vein thrombosis. The patient started anticoagulation therapy with low molecular weight heparin (dalteparin). Initial tumor markers were: beta-choriogonadotropin 46.8 µg/l, alfa-fetoprotein 3.1 U/ml, lactate dehydrogenase (LD) 76.8 µkat/l. Systemic therapy with bleomycin, etoposide and cisplatin (BEP) was commenced (day 1) when the patient’s complaint about acute abdominal pain urged the radiographic re-examination. CTA scan detected mass enlargement in the retroperitoneum, suspecting a rupture of dorsal aortic wall 38 mm above the inferior mesenteric artery origin, with active extravasation and irregular pseudoaneurysmatic dilatation of the aorta below the leak area (Fig. 1). Persisting intratumoral hemorrhage required an endovascular repair procedure of infrarenal abdominal aorta (Fig. 2). Two tubular components *(ETTF2323C70EE*, *ETCF2525C49EE) *of Endurant abdominal stent-grafts were implanted via right common femoral artery and the intervention was performed by specialists at the National Institute of Cardiovascular Diseases in Bratislava. Suprarenal anchoring with not covered stent-like part of abdominal endograft (so called „bare-springs “ with metal hooks) was used, as standard approach for better fixation and sealing, prevention of endoleaks, shifting, or late migration, in case of tubular or bifurcation stentgraft fixation to infrarenal part of abdominal aorta. Dual antithrombotic therapy (acetylsalicylic acid and clopidogrel) was initiated immediately after stentgraft insertion. Postoperative course was uneventful and patient’s performance status allowed for administration of 1. cycle of BEP regimen, 6 days after the endovascular repair surgery.

At the start date of the second cycle of BEP chemotherapy, the patient was admitted for acute intestinal hemorrhage causing severe anemia (G3 according to National cancer Institute—NCI and National Institute of Health—NIH) and circulatory collapse. CTA scan refuted the suspicion of intratumoral bleeding and further examination including the upper gastro-intestinal tract (GIT) endoscopy identified the source of bleeding – 2 duodenal ulcers (Forrest IIb, III). Hemostyptic, antiulcer and vasopressor agents were applied along with the hemo substitution and volume therapy, with positive effect. Within following weeks, two more cycles of BEP were administered until the re-staging CT examination was performed. The retroperitoneal tumor mass shrank to 140 × 110 × 125 mm (CCxLLxAP), still compressing the inferior caval vein (VCI), encasing the aorta and deviating nearby intestinal loops as well as the left kidney. Tumor markers were normalized. Subsequently, the patient underwent left inguinal radical orchiectomy and epididymectomy (and spermatic cord removal). Histologically no vital cancer cells were identified and all specimens consisted of completely necrotic testicular tumor tissue. The patient had been closely observed during a 6-month period after the radical surgery and repeatedly underwent the radiologic evaluation which reaffirmed the ongoing reduction of retroperitoneal formation to 120 × 80 mm. At this point, retroperitoneal lymph node dissection (RPLND) was suggested and performed, associated with the resection of VCI and the left pannephrectomy. Ten days before elective surgery, antithrombotic therapy was changed to low molecular weight heparin in cooperation with hematologists. Doppler ultrasound examination of deep vein system of lower extremities prior to RPLND showed good blood flow in femoral as well as external iliac veins, no signs of thrombosis were present. Bilateral iliac vein thrombosis diagnosed at first presentation of patient resolved completely, and total infrarenal occlusion of inferior caval vein was caused by tumor itself before RPLND. Collateral blood flow was sufficient and therefore, we planned to do no reconstruction after resection. During resection, anesthesiologist referred hypotension short time after clamping of infrarenal caval vein so peroperatively we made the decision to do reconstruction to improve central venous inflow. As there was severe fibrosis on the left side with multiple collaterals, we decided to do just unilateral reconstruction on the right side. Inferior caval vein was reconstructed by using ringed rifampicin-tinctured polytetrafluoroethylene graft (Gore-Tex)—a 10 mm prosthesis was placed between the right iliac vein and VCI. Postoperatively no lower limb edema was present with patent femoral veins and external iliac veins on both sides confirmed by Doppler ultrasound. During surgery heparin at dose 100 units per kilogram was administered, followed by continual intravenous infusion with aPTT levels between 2–3, then changed for full anticoagulant therapy with low molecular weight heparin on postoperative day 10 and 28 days after surgery, dual antithrombotic therapy was restarted.

**Fig. 3:**
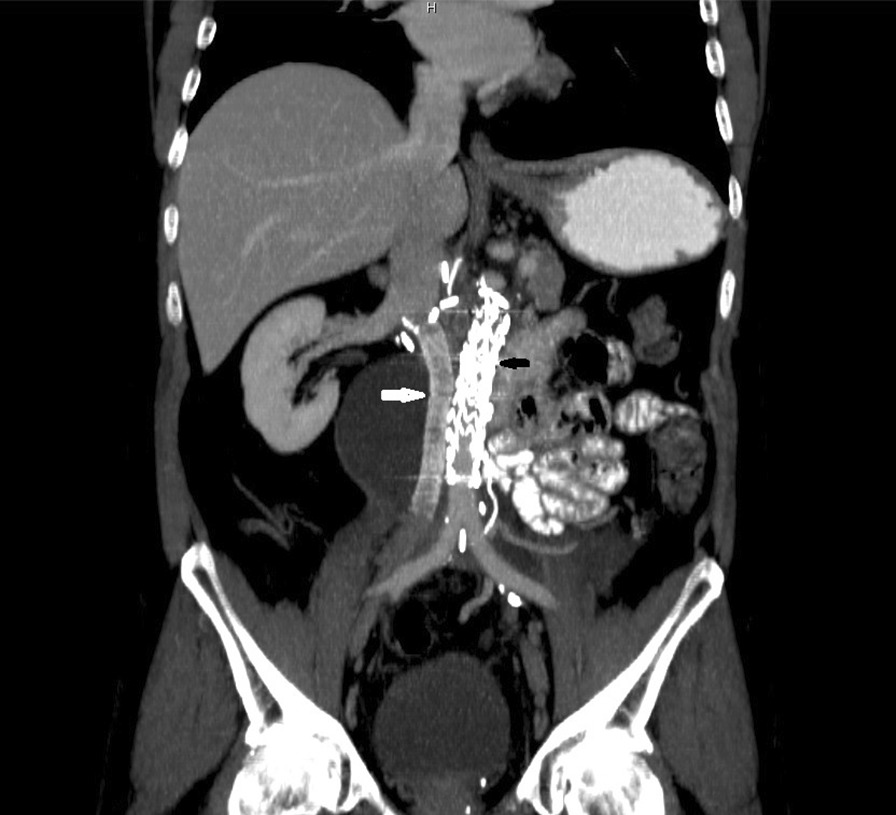
Restaging CT scan (coronal view). Aortic endograft (black arrow), venous PTFE endograft (white arrow)

Surgery duration was 6.5 h, total blood loss estimates 1500 ml, early complications included cardiovascular instability, oliguria and fever. On postoperative day 2, bloody drain output urged an acute CTA scan revealing an extensive perisplenic hematoma, and as the patient became hemodynamically unstable, second look surgery was performed (surgical complication classified Clavien-Dindo grade IIIb). While it failed to identify the origin of bleeding, further postoperative course was uneventful and led to full recovery. Once again, histopathological examination detected no viable cancer structures in the samples acquired from the RPLND, VCI resection and nephrectomy. To this date, 18 months post the final surgical procedure the patient remains disease-free, asymptomatic with maintained patency of the aortic stent graft (Fig. 3).

## Discussion and conclusions

Representing the most frequent malignancy in young men (aged 15–34 years), testicular germ cell cancer patients are fortunate to have a good prognosis [10]. Most of them are diagnosed with stage I disease (confined to the testicle) yet there are effective treatment modalities available to control the disease even in advanced stages. Cisplatin- based regimen followed by surgical removal of residual masses are the cornerstone of successful treatment [11]. A nerve-sparing template RPLND is valid surgical approach in both primary and post-chemotherapy setting. Post-chemotherapy RPLND is recommended in cases of residual CT masses sized 1 cm or more in nonseminomatous germ cell tumors and fluorodeoxyglucose (FDG)—avid masses of 3 cm and more in seminomatous germ cell testicular cancer [12].

A number of additional procedures may be required to reach the complete resection which is an inevitable condition to optimize the clinical outcome. Either anticipated or unexpected secondary surgeries involve mainly caval vein reconstructions (resection, repair), nephrectomy, splenectomy and thrombectomy. Pancreatic and hepatic resections, bowel resection with or without ostomy, thoracotomy and aortic reconstruction (repair) may also be undertaken as part of RPLND [13].

Arterial reconstructions are usually performed by primary anastomosis, reinsertion, or synthetic prostheses, expanded polytetrafluoroethylene (ePTFE) or Dacron. Autologous vein is not routinely used for arterial repair. Aortic resection adjunctive to the post-chemotherapy RPLND is uncommon and is performed either for a nonrepairable injury of the vessel or on behalf of its inclusion in the tumor mass [14, 15]. Herein, previously endovascularily treated aorta was dissected in sub-adventitial layer and aortal resection was not necessary.

Localized defects of inferior caval vein are usually repaired by venoplasty or venous (synthetic) patch while circular damage needs to be reconstructed by either a primary anastomosis, limited by low mobility of remaining ends, or more routinely performed ePTFE prostheses. Veins occluded by a thrombotic process are treated by ligation of their proximal and distal stumps [14, 15]. In our case a circular infiltration of VCI with left common iliac vein obstruction was caused by the tumor itself. Peroperatively, we decided to perform unilateral reconstruction by using the interposed bypass between right common iliac vein and infrarenal segment of VCI.

Tumor infiltration is a rare cause of aortic rupture. Besides the germ cell testicular cancer [16], abdominal aortic invasion has also been reported in other types of malignancies as soft tissue sarcomas [17–20], paragangliomas [21], lymphomas [22] and gynecological tumors [23]. Most authors consent that endovascular aortic intervention is an effective and safe method which should be encouraged to provide a definite therapeutic impact in a subset of patients who would otherwise not be candidates for curative surgery. Radical tumor removal was well proved to diminish the risk of in-field and systemic recurrence and is therefore an ultimate goal of surgical therapeutic approach.

The patient in this report presented with left-sided testicular tumor and bulky retroperitoneal disease compressing the inferior caval vein as well as the left ureter and was indicated for first line chemotherapy treatment when CTA scan revealed an active leak from dorsal aortic wall. Acute endovascular repair was performed in order to assure the feasibility of oncological treatment. Successful upfront endovascular aortic repair in our patient enabled the delivery of systemic oncologic cisplatin-based chemotherapy with good treatment response followed by radical orchiectomy in seronegative disease and subsequent RPLND along with the nephrectomy and caval vein reconstruction.

Aortic infiltration and rupture secondary to germ cell testicular tumor is a rare condition. With respect to the literature, vascular intervention on aorta is rather a part of elective postchemotherapy RPLND. Timely recognition of active intratumoral hemorrhage in metastatic retroperitoneal disease of testicular origin owing to the abdominal aortic rupture enables an appropriate surgical intervention followed by adequate systemic oncologic treatment, and thereby dramatically improves patients’ outlook.

## Data Availability

Data sharing is not applicable to this article as no datasets were generated or analyzed during the current study.

## Abbreviations

AP: Antero-posterior
APTT: Activated partial thromboplastin time
BEP: Bleomycin, etoposid, cisplatin
CC: Cranio-caudal
CT: Computed tomography
CTA: Computed tomographic angiography
ePTFE: Expanded polytetrafluoroethylene
FDG: Fluorodeoxyglucose
GIT: Gastrointestinal tract
IGCCCG: The International Germ Cell Cancer Collaborative Group
LD: Lactate dehydrogenase
LL: Latero-lateral
NCI: National Cancer Institue
NIH: National Institute of Health
RPLND: Retroperitoneal lymph node dissection
VCI: Vena cava inferior
